# Comparative study on the specimen thickness measurement using EELS and CBED methods

**DOI:** 10.1186/s42649-020-00029-4

**Published:** 2020-05-12

**Authors:** Yoon-Uk Heo

**Affiliations:** Graduate Institute of Ferrous Technology, Pohang University of Science and Technology, Cheongam-Ro 77, Hyoja dong, Pohang, 37-673 Republic of Korea

**Keywords:** Thickness measurement, EELS, CBED, TEM, Carbon contamination method

## Abstract

Two thickness measurement methods using an electron energy loss spectroscopy (EELS) and 10a convergent beam electron diffraction (CBED) were compared in an Fe-18Mn-0.7C alloy. The thin foil specimen was firstly tilted to satisfy 10a two-beam condition. Low loss spectra of EELS and CBED patterns were acquired in scanning transmission electron microscopy (STEM) and TEM-CBED modes under the two-beam condition. The log-ratio method was used for measuring the thin foil thickness. Kossel-Möllenstedt (K-M) fringe of the $\mathbf{13}\overline{\mathbf{1}}$ diffracted disk of austenite was analyzed to evaluate the thickness. The results prove the good coherency between both methods in the thickness range of 72 ~ 113 nm with a difference of less than 5%.

## Introduction

Transmission electron microscopy (TEM) as a powerful tool for fine analysis is contributing to the development of advanced materials in material science. The role of TEM is highlighted by characterization on the defect structure and fine precipitate. However, TEM has limitations on the view of statistical and quantitative evaluations due to its small observation area. Despite the limited observation field, TEM has been used to quantify the fraction of small things due to its excellent resolution. Dislocation density is evaluated in TEM (Murr 1970; Willams and Carter 2009; Hirsch et al. 1977). The volume fraction of fine precipitates is also measured through the TEM (Yang et al. 2005; Dorin et al. 2015; Delmas et al. 2004). Although there were the statistical corrections on the prejected dislocation density (Murr 1970; Bailey and Hirsch 1960) and the volume fraction of precipitates (Underwood 1970), the accuracy of those evaluations is related to the exact measurement of thin foil thickness.

Generally, the thin foil thickness is measured using a TEM- convergent beam electron diffraction (CBED) method. Kossel-Möllenstedt (K-M) fringe under two-beam condition is used for the calculation of thin foil thickness. This method is good enough to use broadly in the metallic alloys and the accuracy of measurement is less than 2% (Allen 1981; Kelly et al. 1975). However, K-M fringe is degraded as the dislocation density in the matrix increases by the mechanical deformation. The fine dispersion of precipitate in the matrix hinders the clear identification of K-M fringe. The TEM-CBED method cannot be applicable in the deformed structure and the matrix having fine precipitates.

Thin foil thickness can be measured through the electron energy loss spectroscopy (EELS). Low loss spectra including zero-loss and plasmon peaks are used for the calculation. Regarding the previous report (Egerton and Cheng 1987), this method has an accuracy of less than 2 nm (10%) in the thickness range of 10 to 150 nm. Although this method needs additional information such as convergence and collection angles, the strong benefit where it can be applied regardless of material state promotes the use of this method.

In this study, we aim the reassessment of the relative accuracy of the EELS thickness measurement method for future applications in the deformed metallic materials or fine precipitate-bearing materials. The observed results of EELS and CBED at the coincident positions in an Fe-18Mn-0.7C alloy are compared and further discussed.

## Material and methods

### Specimen preparation and characterization

The ingot of an Fe-18Mn-0.7C alloy was prepared by vacuum induction melting and subsequently hot-rolled to 4 mm thickness. Annealing treatment was performed at 850 °C for 10 min after cold-rolling of 50%. TEM specimen was prepared by electrochemical polishing in a solution of 12 pct perchloric acid + 90 pct acetic acid at room temperature after mechanical thinning to 100 μm thickness. Final Ar^+^ ion milling was conducted to remove the etching effect formed at the electrochemical polishing using a precision ion-polishing system (PIPS, GATAN 691, New York, USA) with the accelerating voltage of 1.0 keV for 20 min. The specimen was observed in a JEOL 200 kV field-emission transmission electron microscope (JEM-2100F) equipped with a Gatan 776 EELS spectrometer (Enfina 1000) under the accelerating voltage of 200 keV.

## Results and discussion

### Thickness measurement using TEM-CBED method

**Fig. 1:**
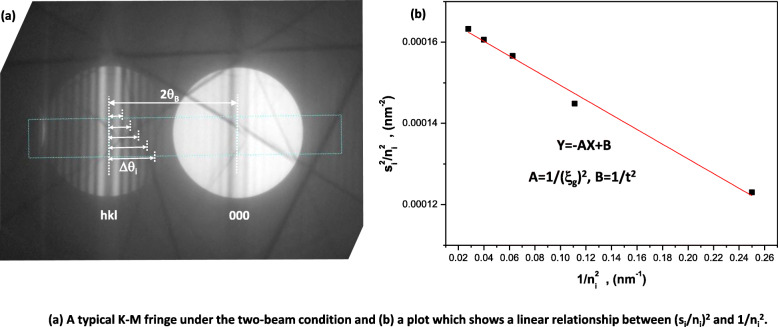
**a** A typical K-M fringe under the two-beam condition and **b** a plot which shows a linear relationship between (s_i_/n_i_)^2^ and 1/n_i_^2^

Following the dynamical theory of diffraction contrast, the amplitude of the diffracted wave (*ϕ*_*g*_) and the specimen thickness (t) have the following relationship (Willams and Carter 2009; Hirsch et al. 1977);

1 $$ {\phi}_g^2={\left(\frac{\pi }{\xi_g}\right)}^2\frac{{\mathit{\sin}}^2\pi t{s}_{eff}}{\pi t{s}_{eff}} $$

where *ξ*_*g*_ is the extinction distance of material and the effective deviation vector *s*_*eff*_ has the relation of ${s}_{eff}=\sqrt{s^2+1/{\xi}_g^2}$ . Equation (1) shows also the relation between a deviation vector and a contrast of the image at the constant thickness. A representative case of that is the contrast modulation in a CBED disk. Figure 1(a) is a typical K-M fringe under the two-beam condition. The intensity minima in the K-M fringe of the diffracted hkl CBED disk can be obtained when *ϕ*_*g*_ *=* 0*.* On the other hand, a deviation vector *s*_*i*_ for i^th^ fringe in a diffracted disk has following relationship (Willams and Carter 2009; Hirsch et al. 1977);

2 $$ {s}_i=\left(\frac{\lambda }{d_{hkl}^2}\right)\left(\frac{\Delta {\theta}_i}{2{\theta}_B}\right) $$

where λ is a wavelength of the electron, d_hkl_ is an interplanar distance of hkl plane, and θ_B_ is the Bragg angle for hkl plane. Combining equation (1) and (2) and applying *ϕ*_*g*_ *=* 0 condition (t × s_eff_ = n_i_, n_i_ is an integer), we can obtain the following equation;

3 $$ \frac{s_i^2}{n_i^2}=-\frac{1}{\xi_g^2}\bullet \frac{1}{n_i^2}+\frac{1}{t^2}. $$

Referring equation (3), we can obtain the specimen thickness using the intercept value in a $\frac{s_i^2}{n_i^2}$ vs. $\frac{1}{n_i^2}$ plot (Fig. 1(b)).

**Fig. 2:**
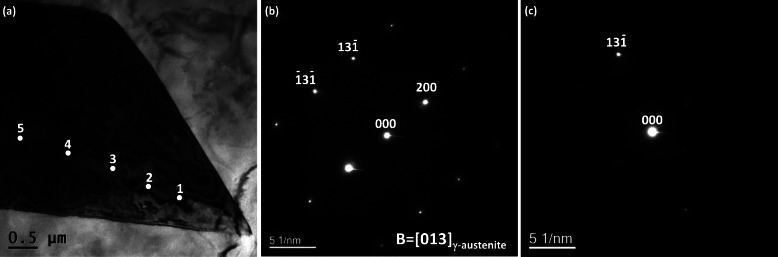
**a** BF-TEM image and **b** corresponding electron diffraction pattern, and **c** the obtained two-beam spot

**Fig. 3:**
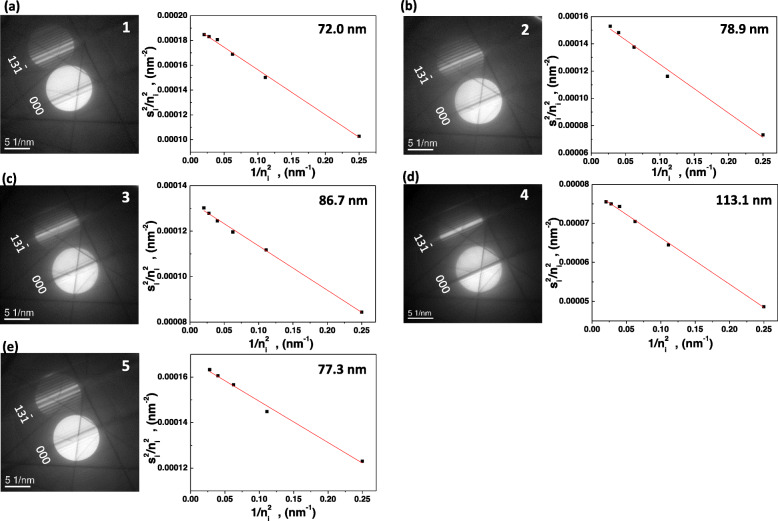
**a**-**e** TEM-CBED patterns and corresponding (s_i_/n_i_)^2^ and 1/n_i_^2^ plots at positions 1 to 5 in Fig. 2(a)

To obtain the K-M fringe, the specimen tilted to the [013] on-axis condition. Bright field (BF) – TEM image and corresponding electron diffraction pattern are shown in Figs. 2(a) and (b). The specimen was tilted from the on-axis condition to obtain a two-beam condition. 000 and $13\overline{1}$ spots show a strong intensity in the obtained two-beam condition (Fig. 2(c)). K-M fringes were obtained at the positions 1 to 5 in Fig. 2(a) as shown in Figs. 3(a) to (e). The specimen thicknesses were calculated by equation (3). Specimen thicknesses show maximum at the position 4 and minimum at position 1 in the range of 72 ~ 113 nm.

### Thickness measurement using EELS spectra

Three different methods (log-ratio method, Bethe sum rule, and Kramers-Kronig sum rule) for the measurement of thickness using an EELS spectrum were introduced in the previous reports (Egerton and Cheng 1987; Egerton 1996). Among them, the current study used the log-ratio method.

**Fig. 4:**
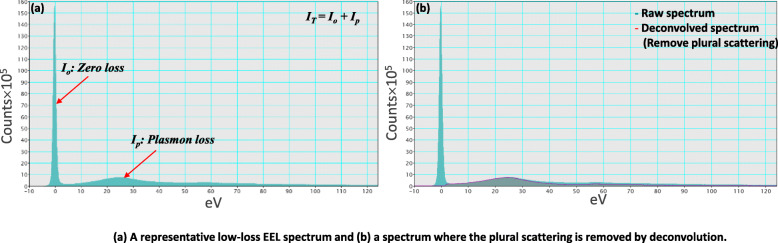
**a** A representative low-loss EEL spectrum and (b) a spectrum where the plural scattering is removed by deconvolution

Figure 4(a) shows a representative low-loss EELS spectrum. The low-loss spectrum includes zero loss (I_0_) and plasmon loss (I_p_) peaks. Specimen thickness is obtained by the following formula (Egerton and Cheng 1987);

4 $$ \mathrm{t}=\Lambda \mathrm{ln}\frac{I_t}{I_0} $$

where Λ is the average mean free path for inelastic scattering of electron and *I*_*t*_ is the total integration of EELS spectra. Λ can be obtained from the following relations;

5 $$ \Lambda \left(\mathrm{nm}\right)=\frac{106F\left(\frac{E_0}{E_m}\right)}{\ln \left(\frac{2{E}_0\beta }{E_m}\right)},F=\frac{1+{E}_0/1022}{{\left(1+\frac{E_0}{511}\right)}^2},{E}_m=7.6{Z}^{0.36} $$

where F is a relativistic factor, *β* is the collection semi-angle in mrad, and *E*_0_ is the incident energy in keV. F, *β*, and *E*_0_ in the current experimental condition are 0.618, 1.3 mrad, and 200 keV, respectively. In the alloy system, atomic number is replaced to the effective atomic number (Z_eff_) which can be obtained by the following formula;

6 $$ {Z}_{eff}=\frac{\sum_i{f}_i{Z}_i^{1.3}}{\sum_i{f}_i{Z}_i^{0.3}} $$

where *f*_*i*_ is the atomic fraction of each element which has atomic number Z_i_. Z_eff_

is calculated to 25.4 in the Fe-18Mn-0.7C alloy. The thickness of the specimen calculated from low-loss EEL spectra after removing plural scattering (Fig. 4(b)) by Fourier-log deconvolution (Egerton 1996).

**Fig. 5:**
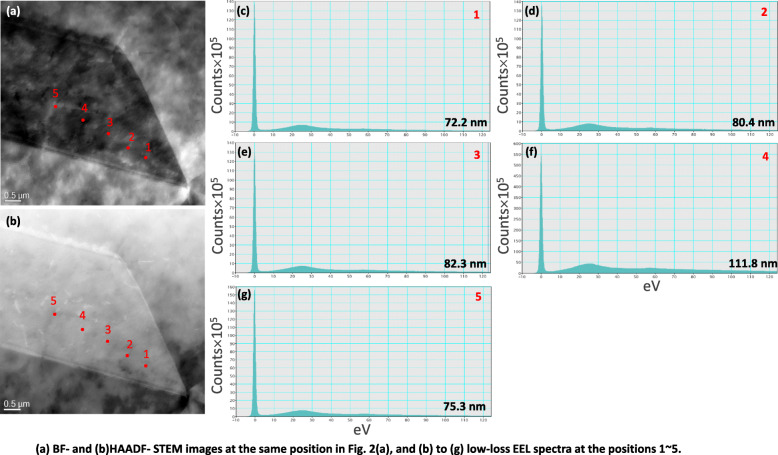
(**a**) BF- and (**b**)ADF-STEM images at the same position in Fig. 2(a), and (b) to (g) low-loss EEL spectra at positions 1 ~ 5

Figure 5(a) and (b) are BF- and high angle annular dark field (HAADF)- scanning TEM (STEM) images in the same area where thicknesses were measured by the TEM-CBED method. The observation is performed under the coincident condition without any tilting of the specimen. Therefore, the measured thickness from the EEL spectrum can be directly compared. The positions (1 ~ 5) in Fig. 5(a) and (b) correspond to the same positions in Fig. 2(a). The obtained low-loss spectra in positions 1 to 5 are displayed in Fig. 5(c) to (g). Applying the experimental parameters which are explained above, we calculate specimen thicknesses at each position using a software (Digital Micrograph 1.8, Gatan Inc., New York, USA). The calculated thicknesses show a similar tendency with those in the CBED method (Fig. 3(a) to (e)); maximum thickness is 111.8 nm in position 4 and minimum thickness is 72.2 nm in position 1.

### Comparison of TEM-CBED and EELS methods

**Fig. 6:**
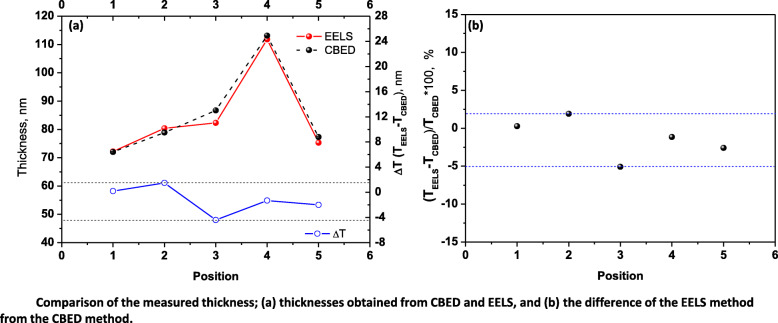
Comparison of the measured thickness; (**a**) thicknesses obtained from CBED and EELS, and (**b**) the difference of the EELS method from the CBED method

**Fig. 7:**
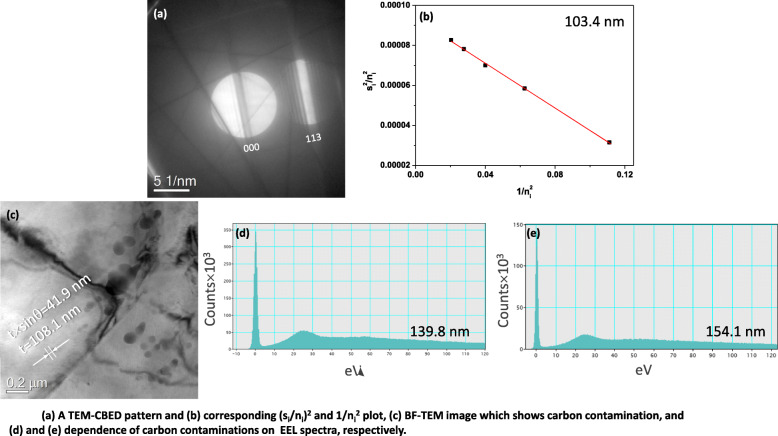
**a** A TEM-CBED pattern and **b** corresponding (s_i_/n_i_)^2^ and 1/n_i_^2^ plot, **c** BF-TEM image which shows carbon contamination, and (**d**) and **e** dependence of carbon contaminations on EEL spectra, respectively

The obtained thicknesses using TEM-CBED and EELS methods are shown together in Fig. 6(a). Both methods reflect well the local thickness variation such as a gradual increase from position 1 to position 4 and an abrupt decrease from position 4 to position 5. The maximum difference between both methods is 4.4 nm at position 3. Comparing the obtained thickness, the CBED method shows slightly higher than the EELS method except position 2. The difference of EELS method from CBED method is evaluated as the form of $\frac{\left({T}_{EELS}-{T}_{CBED}\right)}{T_{CBED}}\times 100$ in Fig. 6(b). EELS method shows the minimum deviation (0.3%) at position 1 and maximum deviation (− 5.1%) from the CBED method at position 3. Even though the measured thickness from the EEL spectrum is similar to that from the CBED method, the carbon contamination on the specimen surface hinders the exact measurement of thickness. Figures 7(a) and (b) are a TEM-CBED pattern and a corresponding $\frac{s_i^2}{n_i^2}$ vs. $\frac{1}{n_i^2}$ plot. The measured thickness is 103.4 nm. EEL spectra were obtained after long exposure to make carbon contamination in the same position. The calculated thicknesses increase from 139.8 to 154.1 nm as the acquisition time of EEL spectra is retarded (Fig. 7(d) and (e)). These values are far from the CBED result. Caution on the carbon contamination is needed to prevent wrong evaluation when the EELS method is applied for the specimen thickness measurement. To see the carbon contamination, the specimen is tilted to 22.8°. As shown in Fig. 7(c), the carbon contaminations on the top and bottom surfaces of the specimen are resolved by two dark spots. A separation distance (r) is measured to 41.9 nm. The specimen thickness is re-evaluated by the following equation (Pan et al. 1994);

7 $$ \mathrm{t}=\frac{r}{\sin \theta } $$

where θ is tilting angle from EELS measurement condition. The measured specimen thickness from the carbon contamination is 108.1 nm. This value is similar to the CBED result in Fig. 7(b). Therefore, the obtained thickness values from EEL spectra in Fig. 7(d) and (e) are too large, and this is caused by carbon contamination.

## Conclusion

Two methods, TEM-CBED and EELS, for thin foil thickness measurement were compared in an Fe-18Mn-0.7C alloy. The EELS log-ratio method shows a good coherency with the TEM-CBED method in the thickness range of 72 ~ 113 nm with a difference of less than 5%. The carbon contamination alters a low-loss EEL spectrum and increases the measured thickness in the EELS method. The specimen thickness was reconfirmed by the measurement of the separation distance between the top and bottom contamination spots. The good coherency between EELS log-ratio and TEM-CBED methods gives an idea of the application of the EELS method on the heavily deformed metallic material where the TEM-CBED method is not applicable.

## Data Availability

The datasets used and/or analyzed during the current study are available from the corresponding author on reasonable request.

## References

[CR1] Allen SM (1981). Foil thickness measurements from convergent-beam diffraction patterns. Phil. Mag. A.

[CR2] Bailey JE, Hirsch PB (1960). The dislocation distribution, flow stress, and stored energy in cold-worked polycrystalline silver. Phil. Mag..

[CR3] Delmas F, Casanove M-J, Lours P, Couret A, Coujou A (2004). Quantitative TEM study of the precipitation microstructure in aluminium alloy Al (MgSiCu) 6056 T6. Mater. Sci. Eng. A.

[CR4] Dorin T, Donnadieu P, Chaix J-M, Lefebvre W, Geuser FD, Deschamps A (2015). Size distribution and volume fraction of T_1_ phase precipitates from TEM images: Direct measurements and related correction. Micron.

[CR5] Egerton RF (1996). Electron Energy-Loss Spectroscopy in the Electron Microscope.

[CR6] Egerton RF, Cheng SC (1987). Measurement of local thickness by electron energy-loss spectroscopy. Ultramicroscopy.

[CR7] Hirsch P, Howie A, Nicholson RB, Pashley DW, Whelan MJ (1977). Electron Microscopy of Thin Crystals.

[CR8] Kelly PM, Jostsons A, Blake RG, Napier JG (1975). The determination of foil thickness by scanning transmission electron microscopy. Phys. Status Solidi.

[CR9] Murr LE (1970). Electron Optical Applications in Materials Science.

[CR10] Pan Z, Davies CKL, Stevens RN (1994). Measurement of foil thickness in transmission electron microscopy. J. Mater. Sci..

[CR11] Underwood EE (1970). Quantitative stereology.

[CR12] Willams DB, Carter CB (2009). Transmission Electron Microscopy.

[CR13] Yang Z, Tirry W, Schryvers D (2005). Analytical TEM investigations on concentration gradients surrounding Ni_4_Ti_3_ precipitates in Ni-Ti shape memory material. Scripta Mater..

